# How can we support best practice? A situational assessment of injury prevention practice in public health

**DOI:** 10.1186/s12889-020-08514-x

**Published:** 2020-04-03

**Authors:** Sarah A. Richmond, Sarah Carsley, Rachel Prowse, Heather Manson, Brent Moloughney

**Affiliations:** 1 Applied Public Health Science Unit, Health Promotion, Chronic Disease and Injury Prevention, Public Health Ontario, 480 University Ave, Suite 300, Toronto, ON M5G 1V2 Canada; 2 Dalla Lana School of Public Health, Division of Epidemiology, University of Toronto, Toronto, ON Canada; 3 Health Promotion, Chronic Disease and Injury Prevention, Public Health Ontario, Toronto, ON Canada; 4 Dalla Lana School of Public Health, Clinical Public Health Division, University of Toronto, Toronto, ON Canada; 5 School of Public Health and Health Systems, University of Waterloo, Waterloo, ON Canada

**Keywords:** Situational assessment, Public health, Injury prevention, Capacity building, Best practice

## Abstract

**Background:**

To effectively impact the significant population burden of injury, we completed a situational assessment of injury prevention practice within a provincial public health system to identify system-wide priorities for capacity-building to advance injury prevention in public health.

**Methods:**

A descriptive qualitative study was used to collect data on the current practice, challenges and needs of support for injury prevention. Data was collected through semi-structured interviews (*n* = 20) and focus groups (*n* = 19). Participants included a cross-section of injury prevention practitioners and leadership from public health units reflecting different population sizes and geographic characteristics, in addition to public health researchers and experts from academia, public health and not-for-profit organizations. Thematic analysis was used to code all of the data by one reviewer, followed by a second independent reviewer who coded a random selection of interview notes. Major codes and sub codes were identified and final themes were decided through iterations of coding comparisons and categorization. Once data were analysed, we confirmed the findings with the field, in addition to participating in a prioritization exercise to surface the top three needs for support.

**Results:**

Major themes that were identified from the data included: current public health practice challenges; capacity and resource constraints, and; injury as a low priority area. Overall, injury prevention is a broad, complex topic that competes with other areas of public health. Best practices are challenged by system-wide factors related to resources, direction, coordination, collaboration, and emerging injury public health issues. Injury is a reportedly under prioritized and under resourced public health area of practice. Practitioners believe that increasing access to data and evidence, and improving collaboration and networking is required to promote best practice.

**Conclusions:**

The results of this study suggest that there are several system level needs to support best practice in public health injury prevention in Ontario including reducing research to practice gaps and supporting opportunities for collaboration. Our research contributes to the literature of the complexity of public health practice, and presents several mechanisms of support to increase capacity at a system level to improve injury prevention practice, and eventually lessen the population burden of injury.

## Background

Injury represents the leading cause of morbidity and mortality for Canadians ages 1–34 years [1]. It is estimated that every hour, over 400 Canadians suffer a preventable injury that requires medical attention [1]. This represents over 9500 people seen in emergency rooms, 600 hospitalized, and 40 deaths, on a daily basis [1]. For many of those who suffer a serious injury, there are lifelong sequelae including reductions in physical activity, the inability to work, and poorer quality of life. Injuries cost the health care systems in Canada an estimated 27 billion dollars annually, and is increasing [1]. From 2004 to 2010, the cost of injury increased by 35% and is estimated to increase to $75 billion a year, given the current trajectory [1]. It is important to address the burden of injury in Canada, particularly given the predictable and preventable nature of the majority of injuries. The burden of injury can be reduced by implementing evidence-based population-level interventions [2].

In the province of Ontario, Canada there are 35 locally governed public health units serving a combined population of 14.4 million [3] representing over one-third of all of Canadians [4]. A set of provincial Ontario Public Health Standards (OPHS) establish the requirements for public health action including a comprehensive health promotion approach to the prevention of a range of injury types [5]. This includes consideration of: concussions; falls; suicide; road and off-road safety; and violence. To inform public health intervention planning, practitioners need to integrate information on: the incidence and prevalence of injury; the risk and protective factors for their occurrence; stakeholder perspectives; existing programs and services; and evidence of effective interventions. In addition, there is a need to know how to implement and evaluate interventions. These tasks are challenging for any one organization or individual to undertake with any level of efficiency or effectiveness.

There is substantial review-level literature that supports the need for public health practice to implement evidence-based interventions [6–9]. Despite awareness of and intention to use evidence-based interventions, research suggests that their use is limited [7]. This may be due to the lack of capacity in knowledge and skills to select, adapt, and implement appropriate interventions [7]. Previous research has shown that public health practitioners can be supported to make evidence-based or -informed decisions by the use of tools, training, scientific and technical assistance, and quality assurance and improvement [9]. However, literature suggests that the variations that exist in the practice context should be understood before this capacity building can occur [7]. In addition, it is necessary to tailor capacity-building strategies to address these variations to have wide-reaching and lasting impact [7].

Capacity building within and across the health system requires support across multiple sectors including those that provide governance, funding, data and data systems, and monitoring and evaluation [8]. In the context of injury prevention in Ontario, there are few organizations dedicated to support the evidence-based interventions in the public health system. Public Health Ontario (PHO) is an agency of the provincial government that has a mandate to provide the scientific and technical advice and support to the field of public health [10] and can play an important role in building capacity for evidence-based interventions in public health, including injury prevention.

## Methods

### Aim

The aim of this project was to complete a situational assessment of injury prevention practice across a provincial public health system. In accordance with our (PHO) organizational mandate and role in providing centralized injury prevention expertise, the specific objectives for this project were: (i) to identify priorities for scientific and technical support for public health practice in injury prevention on a system-wide basis; (ii) to gain a better understanding of the current practices and resources across injury prevention topics, and; (iii) develop relationships with public health unit staff and stakeholders to increase opportunities for collaboration and networking. The results specific to objective one are presented here.

### Study design and setting

We used a qualitative descriptive study [11] in the context of injury prevention public health practice in the province of Ontario. Public health practitioners develop a course of action with guidance from the Ontario Public Health Standards (OPHS), [12] with specific use of the Injury Prevention Guideline [5]. The standards identify minimum expectations for programs of public health which includes using of both local data and evidence of best practice to inform programming. In addition, the program plans are informed by consultation and collaboration with the field and other stakeholders to determine program relevance, to build upon existing opportunities, and decrease duplication.

### Data collection and participants

**Fig. 1:**
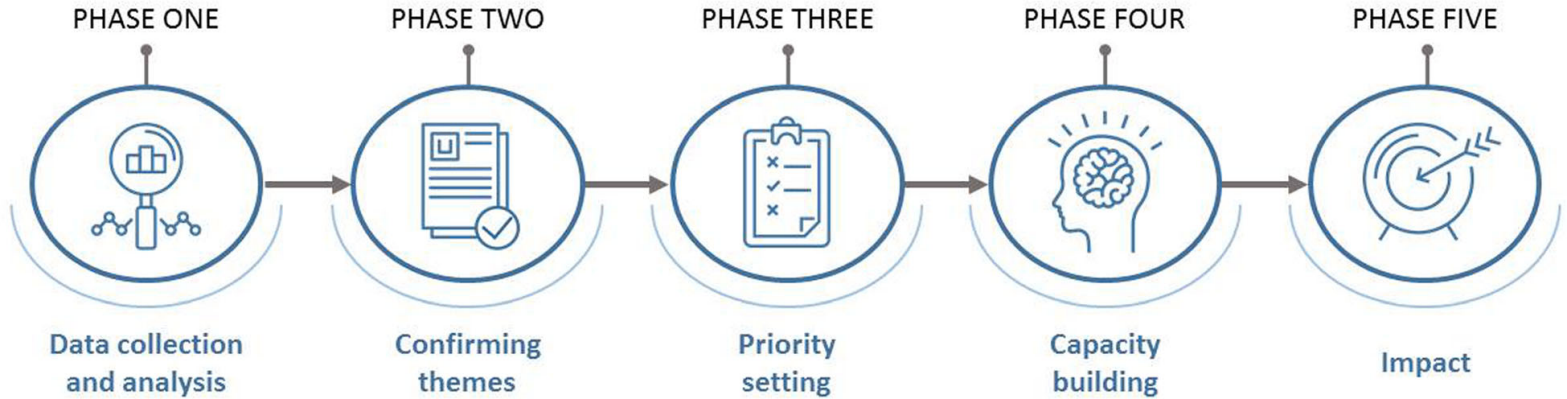
Process model used to guide situational assessment development

Twenty individual key informant interviews and 5 public health unit site visits with 19 focus groups were conducted between January and April, 2018. We used a concurrent data collection and analysis process [11] for this project, beginning with the development of a process model to guide the project across a series of phases (Fig. 1). Data were collected through key informant interviews and focus groups. See Supplementary Table 1 for the key informant and focus group interview guide. A variety of written documents including the provincial standards, guidelines, surveillance data, evidence briefs, and other documents collected from specific units (i.e., those that provided documentation or those that could be attained from public sites) were not analyzed but reviewed to gain a contextual understanding of the work in injury prevention, to better understand the type of interventions that are implemented in public health, and to inform data collection.

We used purposive snowball sampling, acceptable for use with a qualitative descriptive approach [11] for both the key informant interviews and the site visits to collect multiple perspectives across public health practice. This approach identifies key participants, those who know most of the phenomenon of interest [11]. Key informant participants were recruited by email and included injury prevention practitioners and managers in health units, researchers, and not for profit organizations. Public health units selected for site visits reflected different population sizes and geographic characteristics. Focus groups were organized based on role into: front-line injury prevention practitioners, program managers/directors, and medical officers of health (and/or associate medical officers of health). One participant from each of the site visit units (*n* = 5) participated both as a key formant and a focus group participant. Each interview and focus group was approximately 1 h in length and was conducted by the first author, trained in facilitation, and experienced in focus group data collection and analyses. Data were collected by the interviewer and/or a note-taker scribing conversations during the key informant interviews and focus groups.

Open-ended questions were asked in both the semi-structured key informant interviews and focus groups. Questions were framed to invite discussion about the current practice of injury prevention in public health, the existing strengths, challenges and needs for impactful practice (Supplementary Table 1).

### Informed consent process

The need ethics approval and participant consent, written or verbal was waived by the Public Health Ontario Ethics Review Board (PHO – ERB) for this project. The activities described here are outside of the scope of ERB review according to the Tri-Council Policy Statement 2 (2014), Article 2.5: “Quality assurance and quality improvement studies, program evaluation activities, and performance reviews, or testing within normal educational requirements when used exclusively for assessment, management or improvement purposes, do not constitute research for the purposes of this Policy, and do not fall within the scope of research ethics review.” [13] Any information pertaining to the data collection as well as storage and use was provided to participants by direct oral communication. In addition, information on the aims of the study and the rationale for the research was shared with all participants. Throughout the process, participants were invited to provide feedback and to ask questions related to any aspect of the project. All measures of security and anonymity were communicated to all participants ensuring the data collection did not include individual identification.

### Data analysis

Data that were collected from both the key informant interviews and focus groups were used in an iterative thematic data analysis [14]. One reviewer coded all the data independently and a second independent reviewer coded a random selection of interviews and site visits. Major codes and sub codes were identified by both reviewers and any variations in coding application were discussed. Discrepancies in codes and sub codes were resolved via consultation with a third project team member and decisions on a final codebook were made referring to the original interview notes. Data was recoded again using the final codebook. Final themes that were identified from the data were decided through the comparison and categorization of the codes and sub codes. Data analyses were performed concurrently with data collection, allowing the addition of new codes as well as creating space for reflection and data saturation. Once a strong understanding of the context was identified, and there were no additional codes or sub codes emerging in subsequent data collection and analyses, recruitment ceased and data collection was considered complete.

### Data verification strategies

This work included data collection and analysis methods coherent with a qualitative descriptive design [11]. This included concurrent data collection and analyses as well as checking in with our participants across the process to verify what was heard. This iterative process also served to fulfil our third objective, to develop relationships with public health unit staff and stakeholders to increase opportunities for collaboration and networking. All participants were invited to engage in an exercise to confirm the themes that were identified from the analysis. Public health unit staff that did not participate in the data collection were also invited with the goal to collect a variety of perspectives, in addition to ensuring the data gathered reflected the perspectives of those that did not participate. To ensure meaningful conversation, we hosted an online meeting with support from a senior program specialist trained in group facilitation and limited the webinar participation to one representative from each public health unit. Separate follow-up meetings were conducted to share the findings with both the researchers, and not for profit organizations. Themes, challenges, or system needs that participants believed to be missing from the preliminary findings were given consideration to be added to the data. Finally, we used the Consolidated criteria for Reporting Qualitative Research checklist (COREQ) [15] as guidance for reporting these data. See Supplementary Table 2 for the COREQ checklist criteria.

To identify priorities for action, we used prioritization criteria (i.e., impact potential, existing opportunities, and feasibility) selected from the literature [16] to rank the identified system needs (see Table 1). The criteria were selected from a published framework that determined the most relevant criteria for use in decision making for those that aim to support injury prevention practice [16]. A sub-set of the first meeting participants volunteered to participate and were given the list of the needs, in addition to the criteria, 2 weeks before the exercise took place. A live, online ranking prioritization process was conducted where participants discussed and then ranked the needs according to their relative importance based on the three criteria. After discussion, a final prioritization exercise was completed to achieve consensus through group discussion and live ranking on the top three needs for support and action.

**Table 1:** Criteria^a^ for use in priority setting for system level injury prevention support

Criteria	Explanation
1. Impact	• This criterion considers the potential of each option to make an important impact on injury work and injury prevention in Ontario. ***Example: Would this make a significant difference in my injury prevention work, and therefore toward injury prevention in the province?***
2. Existing Opportunities	• The existing opportunities criterion is used to describe any existing efforts to mobilize the need. This could be efforts nationally, provincially, and/or locally. ***Example: Would work on this need build upon existing efforts already underway?***
3. Feasibility	• This criterion describes the feasibility of the need being addressed in a timely manner, or to certainty in the ability of the need to be actioned. ***Example: Can we actually get this done?***

^a^Adapted from: Chambers and Richmond et al. (2016)

## Results

Seventy-one individuals participated in either a key informant interview or focus group. There were no participants that refused to participate. Key informants included public health practitioners and managers (*n* = 12), injury researchers with public policy expertise (*n* = 2), a public health expert (*n* = 1), and not for profit organizations aimed to support injury prevention both provincially (*n* = 2) and nationally (*n* = 3). Site visits included 5 public health units across the province: two representing predominantly rural areas – one being in northern Ontario, two from predominantly urban areas, and one public health unit that provided service to both rural and urban areas. The total number of participants, as well as their positions/roles are shown in Table 2.

**Table 2:** Key informant and focus group participant characteristics

**Participant**	**Sector**	**N**
Public Health Nurse	Public Health	27
Manager/Director	Public Health	22
Medical Officer of Health/Assistant Medical Officer of Health	Public Health	8
Health Promoter/Analyst	Public Health	6
Injury Prevention Practitioner	Not-for-Profit Organizations	5
Injury Prevention Researcher/Public Health Scientist	Academia	3
**TOTAL**		71

Other data collected for this project (e.g., existing provincial standards and guidelines, surveillance data, etc.) provided contextual information for objectives 2 and 3, and are not reported here but provided important information for meaningful discussion of the results.

The major themes that were identified from the data included the current practice challenges in injury prevention, capacity and resource constraints of individuals and the organization, and injury as a low priority area in public health. Although distinct themes, there are relationships between findings across themes which contribute to a more complete picture of the public health practice in injury prevention in Ontario. Within each theme, needs for effective practice were expressed that created opportunity to present areas of potential support. The needs are discussed within each theme, and summarized in Table 3.

**Table 3:** Needs and areas of potential support for injury prevention practice as expressed by participants, by identified theme

**Theme**	**Sub-theme**	**Expressed Needs**	**Areas of Potential Support**
Current Public Health Practice Challenges in Injury Prevention	Collaboration and Networking	• Need for increased collaboration with other public health units • Need for collaboration with research and local and national research networks • Need for continued networking for public health practitioners in injury prevention	• Explore the possibility of housing all injury resources in one place • Assist with a process and framework to develop, enhance, or amalgamate existing networks to best support learning and coordination of efforts toward injury prevention work
Capacity and Resource Constraints	Access to Local Data and Indicators	• Need for access to local data • Need for capacity in analyses and interpretation of data • Need for systematic use of population level indicators • Need for updated population level indicators • Need for programmatic indicators for use in evaluation	• Collaborate with local/regional/provincial data holders to explore access and use of data • Provide recommended population level and programmatic level indicators for injury prevention
	Evidence Summaries	• Need for evidence summaries for risk factors across injury topics • Need for evidence summaries on effective interventions • Need for emerging evidence in injury prevention practice locally, nationally, and internationally	• Survey existing synthesis work and gaps across injury types and scope a plan to address needs across injury topics • Bridge the research to practice gap for emerging injury issues
Injury Prevention as a Low Priority Area	Competing Priorities with other Health Topics	• Need for injury topics to be prioritized for data analysis • Need for local/regional/provincial data and evidence to improve prioritization of injury prevention	• Provide data visualizations for priority injury topics at the provincial and local level • Develop resources to support the process of using data and evidence to inform priorities for injury prevention

### Current public health practice challenges in injury prevention

Current public health practice in injury prevention is challenging due to competitive and limited resources, insufficient direction and coordination, collaboration complexity, and emerging injury issues to address. Injury prevention in Ontario includes many passionate and committed practitioners who described injury as a broad practice area that includes several injury topics (e.g., road safety, off-road safety, falls, and violence). Injury prevention cross cuts and competes with other topics, (e.g., requirements to address the opioid crisis as well as cannabis use). Participants spoke of practitioner’s challenges to fulfil expectations described in the OPHS, citing inadequate resources as a significant barrier to effective practice. Key informants identified insufficient system level guidance and coordination, and inconsistent implementation of programs across the province. Interestingly, there was also a perception that larger public health units in the province would be spared the challenges of smaller units, including those related to the availability of resources and capacity; however, there was little variability in the data between large and smaller units.

Participants stated that collaborative work was challenging. Specifically, they reported difficulty building partnerships and collaborating locally (e.g., many informants described the inability to form collaborative working relationships with sectors outside of health) as well as with other public health units across the province. Participants expressed a gap in knowledge of current research in injury prevention, describing limited knowledge of the research projects happening locally, provincially, and nationally. Practitioners wished for increased connection between the field and injury researchers.

Finally, when asked about the current and emerging issues in injury practice, the following topics were described: i) concussion prevention in the context of sport; ii) the role of public health in older adult falls prevention; and, iii) the need to address cannabis use in the context of impaired driving. These topics were not surprising given the implementation of Rowan’s Law (provincial legislation to support concussion detection, management and prevention) [17] in addition to recent federal legislation in Canada, legalizing the use of cannabis [18]. When addressing current or emerging issues, multiple stakeholders described the potential for “duplication of efforts” across injury topics, specifically where health units are independently summarizing the evidence of effective interventions from the peer-reviewed literature when there is little to no variation in their implementation contexts.

### Capacity and resource constraints

Capacity and resource constraints were described in relation to the lack of both financial and human support for prevention practice overall, and for injury specifically. Sub-themes related to both individual and organizational capacity. Many of those that work in public heath injury prevention are trained public health nurses; however, participants described a large variability in the capacity of individual practitioners. Some discussed the inability to conduct and/or interpret data analyses as well as to critically appraise and synthesize evidence to inform decision making. Many described the lack of time as a significant factor in the ability to do quality analyses and synthesis work; others described confidence and experience, in addition to a skill set in using this information in planning. There was also a stated lack of organizational capacity that included few staff responsible for addressing the breadth of injury topics (i.e., road and off road safety, sport injury, falls, violence, burns, poisoning, drowning) in addition to the support need to adopt, implement and evaluate new and existing evidence-based interventions.

Across all levels of practice, participants described the lack of access to local data to inform planning and programming for injury. While health administrative data is available (e.g., emergency department visits, hospitalizations, deaths) there is variation in the accessibility and use of other types of pertinent data (e.g., police reported collision data). Some public health units have relationships with data holders where others described the inability to develop these relationships entirely. Informing a plan to prevent injury, across all injury types, is reliant on data that provides information on injury type, mechanism, location, severity, as well as demographic information of those involved.

There was a shared concern over the use and availability of both population level and programmatic indicators in injury prevention. The staff spoke of the lack of specificity in administrative data that can be used to establish an understanding of burden, as well as for use in planning a program of public health action. In addition, it was expressed that there is a lack of specific indicators to accurately measure the effect of the work done in public health.

### Injury prevention as a low priority area

Injury prevention was described as a low priority area in public health. Practitioners used the terms “low”, “lowest” or “competing” priority for injury. There was a feeling of significant mismatch between the burden of injury and its impact on the health of Ontarians and the resources available to address it. Stakeholders described that injury topics “sit in other departments” such as the built environment (e.g., safe and active travel), and family health (e.g., child poisoning or safe home practices for child falls prevention) creating a feeling of disconnection with the work being done in the same public health unit. The stakeholders also described the high rate of staff turnover and lack of senior leadership support compared to other health topics.

Public health staff also spoke of injury as a topic that is not prioritized for data analyses at a local level. For example, there is often one epidemiologist in a public health unit department (and sometimes in the entire organization) that is charged to pull and analyse health administrative data across all health topics; in the context of emerging public health issues (e.g., opioid-related morbidity and mortality), injury analyses are deemphasized.

### Data verification and setting priorities

Participants of the online meeting (*n* = 22) and those from each in person meeting (researchers, and not for profit organizations) (*n* = 8) provided confirmation of the themes. No new themes were added to the data collection from information collected in the meeting. From the prioritization exercise, the field ranked the top three needs that included: the need for evidence of effective interventions across injury topics; the need for access to local data and systematic use of both population level and programmatic indicators, and; the need for increased collaboration and networking opportunities both between public health units and research.

## Discussion

The aim of this project was to complete a situational assessment of injury prevention practice across the Ontario public health system. To allow for meaningful unpacking of our data for use in discussion of what can be done to support public health practice in injury prevention, the interpretation of our results was informed by the framework developed by Leeman et al. (2017) [7]. Leeman et al. (2017) developed a theory to characterize the important differences in the practice context of implementing interventions as well as the important variations in the interventions themselves.

The themes that were identified from the data related to the current public health practice challenges of injury prevention, capacity and resource constraints, and injury prevention as a low priority area in public health practice. There are considerable gaps in the availability, access, and knowledge of emerging issues in injury prevention research at a practitioner level, in addition to the need for greater connection between research and practice. Injury prevention practitioners’ ranked increasing access to data and evidence, and improving collaboration and networking as priority needs to influence their practice.

These findings are not surprising given the existing literature that describes the need for increased capacity to fulfil the requirements of public health roles generally, [7, 19–21] as well as across other public health topics including nutrition, [22, 23] mental health, [24] and infection prevention [25]. Further, other situational assessments conducted at PHO in public health nutrition and healthy growth and development revealed consistent challenges including the need for increased collaboration, access to evidence and local data, as well as capacity and resource constraints (unpublished). In addition, the recent report from the Ontario Auditor General states that there is a need for a systematic approach to prevention, as well as increased access to local data, evidence and indicators [26]. Our findings combined with this previous literature highlight the need to create effective system-wide solutions.

The first prioritized item described the need for evidence of effective interventions for public health practice. Leeman et al. (2017) describes two concepts related to the difficulty in implementing evidence-based interventions: complexity and uncertainty [7]. The present study revealed both issues. Uncertainty is created when there is a lack of an evidence base for use in planning [7]. We found that there were gaps in the availability of evidence and the relevancy of evidence-based effective interventions. For example, there is a current gap in evidence of effective interventions to address off-road safety in the literature, particularly from a Canadian perspective [27]. In this case, public health practitioners would focus on increasing awareness of the risk and protective factors, as well as engaging with stakeholders and communicating with local off road vehicle communities to better understand the problem, instead of supporting the implementation of an intervention. The other issue is where there is a breadth of evidence to support prevention, but it may lack relevancy for the role of public health staff. An example of this is in older adult falls prevention where there is a breadth of systematic review level evidence to support the implementation of exercise interventions and medication adjustment programs to reduce the incidence of falls [28]; however, public health practitioners in our study described the challenge to use this individual-focused, clinical research in their practice since these interventions are implemented by other organizations/sectors.

In interventions with a high level of complexity, implementation exists within a context of an increased number of, diversity within, and interdependence of stakeholders, organizations, and system-level support [7]. Data from our study suggests this level of complexity exists for injury prevention. For example, within the portfolio of road safety, there is significant literature supporting effective built environment interventions to reduce collisions using a safe systems approach [29]; however, public health practitioners in our study spoke to the complexity of supporting interventions where decision makers sit across multiple sectors. For example, decisions supporting the implementation of speed humps or lowering speed limits in areas with high pedestrian traffic such as school zones can occur with involvement from the traffic safety division of law enforcement, municipal traffic services, and school board sectors; however, the level of influence that each of these sectors has in the decision-making process varies significantly across Ontario.

Participants highlighted the need for external support to provide evidence summaries, either in the form of literature reviews, rapid reviews, or systematic reviews. Practitioners reported lack of time and capacity to conduct evidence syntheses themselves, given a context of resource constraints and leadership to guide the process. In this case, increasing collaboration with researchers and public health units on areas of specific need for evidence and then providing this information would have a significant impact in the work of public health. In the collaboration process, efforts should be placed in better understanding the type of knowledge product needed, as large-scale systematic reviews of interventions are often not required to make decisions for a program of public health. Literature in this area suggests that using a hierarchical approach, where the use of evidence is strategic, maximizes the impact of injury prevention efforts [16]. It is suggested that highly synthesised, evidence-based strategies should be prioritized for use in planning, in addition, attention should be paid to the important evidence gaps for future research [16].

The second prioritized need described the value in increasing access to local data and systematic use of both population level and programmatic indicators. Data is used to develop locally driven, prevention plans that meet the requirements outlined in the OPHS [5]. The administrative data sources available in Ontario do not provide the specific information to develop fulsome prevention plans, given the lack of injury mechanism, location, and other equally important contextual factors. Further, the existing injury indicators that are populated from these data sources, lack relevancy for evaluation purposes. Many participants described the need to develop programmatic indicators to best represent the work of public health. Given the mandate of PHO to provide scientific guidance to the field, support at a system level is the mechanism of most significant impact. A recent systematic review of the effectiveness of capacity building interventions in public health recommended efforts toward system level intervention given the variability of effectiveness of individual level capacity building [30]. In this case, pursuit of universal access to local data, increasing the usefulness of existing data sources and exploring data sharing agreements across sectors (such as transportation) can provide the necessary inputs to the development of local injury prevention programs. To meet this need requires multi-sectorial, system-level support to improve injury prevention in Ontario.

Finally, this study highlighted a need for improved and on-going collaboration and networking with research and other public health units. The public health unit staff, particularly at the level of front line practitioners, discussed the importance of collaboration and networking equally to the need for access to local data and evidence. This is supported by literature, [7, 31] as well as highlighted in the Public Health Agency of Canada’s Best Practices Portal, where a key element of the public health approach to prevention includes collaboration across sectors and levels that calls for shared responsibility in prevention [32]. Despite much activity related to injury prevention across numerous networks, practitioners described the need to increase collaboration to reduce the duplication of efforts across the province. Further, there is a need for guidance to increase collaboration with local organizations and other community groups, to better understand and influence the applicability of public health programming.

### Strengths and limitations

There are several strengths to this work. To our knowledge, this is the first situational assessment of injury practice across the Ontario public health system. Only through fulsome understanding of the context of practice can needed supports to improve population level outcomes be identified. Data collection and analysis were performed concurrently, allowing the addition of new codes as well as creating space for reflection during the data collection process. Our sample size was relatively large, enhancing the reliability (i.e., duplication) within our data. Our sampling methodology identified the best people to talk to and created opportunities to develop relationships with public health unit staff and stakeholders as well as to develop mechanisms for prolonged engagement with the field of injury prevention. The semi-structured nature of the questions asked during the interviews and focus groups allowed the process to be guided by what the participants considered important. Finally, the iterative process to our methods allowed simultaneous reflection of the literature and the collection and analyses of the data.

There are limitations to this study. First, we were unable to capture every perspective of injury prevention practice across the province. Therefore, it is possible that certain views are not represented in these data and could have further informed both the analyses and prioritization of action. In addition the results of this study may not necessarily be generalizable to other provinces in Canada. Given the nature of our data collection processes, we did not include evidence of participant views on each theme. This may undermine the validity of the data as well as provide a less rich description of the phenomenon of study; however, we had a relatively large sample size, met theoretical saturation and implemented a process of data verification with our participant’s throughout the process of both data collection and analyses. These methods increase the trustworthiness and interpretation of data as presented. Another limitation is of the qualitative descriptive approach itself. This approach stays close to the data and does not abstract far as to generate a theory [11]. Our aim; however, was to provide an accurate account of the context and challenge of injury prevention practice in Ontario. Finally, there is a possibility of researcher bias given the nature of our role at PHO. In this case, we included a second, independent reviewer in our iterative approach to developing a final coding scheme that was applied to each key informant interview and all focus group data.

## Conclusions

The results of this study suggest there are several challenges in injury prevention and opportunities for system-level solutions to support practice in Ontario. We assessed current injury prevention practice, the existing challenges, gaps and needs, and proposed system-level solutions. This includes increasing universal access to local data, providing synthesized evidence across the public health approach, developing applied public health research projects, and creating opportunities for provincial collaboration and networking for and with communities of practice. These efforts can increase the success of injury prevention programming at a local level by reducing the duplication of efforts and by increasing practitioner capacity. Leeman et al. (2017), Hanson et al. (2012) as well as Mitchell and Ryder (2020) describe the need to readdress the public health approach to prevention to include reviewing the necessary inputs to local practice to create effective and sustainable prevention programming [7, 33, 34]. Hanson et al. (2012) describes this need, specific to injury prevention across three main gaps: the research to practice gap; the efficacy to effectiveness gap; and the injury prevention to safety promotion gap. Our results will be used to inform a plan of action to address the needs expressed by practitioners, using our prioritized results, in addition to leveraging existing work across the province. For example, we aim to impact the existing gap in research to practice in injury prevention through applied public health research projects that focus on a context-driven, bottom-up approach [34]. This includes support from those with research and content expertise across injury prevention topics, in addition to those that have expertise in capacity building support in a public health context. Creating system-wide support to advance effective and evidence-based injury practice can have a meaningful impact on reducing the population burden of injury in Ontario and Canada.

## Supplementary information

**Additional file 1: Table S1.** Key informant and focus group interview guides.

**Additional file 2: Table 2.** Consolidated criteria for reporting qualitative studies (COREQ) checklist^1^.

## Data Availability

The datasets used and/or analysed during the current study are available from the corresponding author on reasonable request.

## Abbreviations

OPHS: Ontario Public Health Standards
PHO: Public Health Ontario
PHO-ERB: Public Health Ontario Ethics Review Board
COREQ: Consolidated criteria for Reporting Qualitative Research checklist

## References

[1] Parachute (2015). The Cost of Injury in Canada.

[2] Richmond SA, D'Cruz J, Lokku A, Macpherson A, Howard A, Macarthur C (2016). Trends in unintentional injury mortality in Canadian children 1950–2009 and association with selected population-level interventions. Can J Public Health.

[3] Ministry of Finance (2019). Ontario Fact Sheet.

[4] Statistics Canada (2018). Canada's population estimates, fourth quarter.

[5] Ministry of Health and Long-Term Care (2018). Ontario Public Health Standards. Injury Prevention Guideline.

[6] Katz JW, A. (2016). Technical assistance to enhance prevention capacity: A research synthesis of the evidence base. Prev Sci.

[7] Leeman J, Calancie L, Kegler MC, Escoffery CT, Herrmann AK, Thatcher E (2017). Developing theory to guide building practitioners' capacity to implement evidence-based interventions. Health Educ Behav.

[8] Nores M, Fernandez C (2018). Building capacity in health and education systems to deliver interventions that strengthen early child development. Ann N Y Acad Sci.

[9] Wandersman A, Chien VH, Katz J (2012). Toward an evidence-based system for innovation support for implementing innovations with quality: tools, training, technical assistance, and quality assurance/quality improvement. Am J Community Psychol.

[10] Ontario Government. Ontario Agency for Health Protection and Promotion Act, Chapter 10 (Schedule K). Ottawa, ON; 2007.

[11] Sandelowski M (2000). Whatever happened to qualitative description?. Res Nurs Health.

[12] Ministry of Health and Long Term Care. Ontario Public Health Standards. Requirements for Programs, Services, and Accountablity. http://www.health.gov.on.ca/en/pro/programs/publichealth/oph_standards/. Accessed: 12 May 2019.

[13] Canadian Institute of Health Research, Natural Sciences and Engineering Research Council of Canada, and Social Sciences and Humanities Research Council of Canada (2014). Tri-Council Policy Statement: Ethical Conduct for Research Involving Humans.

[14] Braun V, Clarke V (2006). Using thematic analysis in psychology. Qual Res Psychol.

[15] Tong A, Sainsbury P, Craig J (2007). Consolidated criteria for reporting qualitative research (COREQ): a 32-item checklist for interviews and focus groups. Int J Qual Health Care.

[16] Chambers A, Richmond SA, Logan L, Macarthur C, Mustard CA (2015). The development of a framework to integrate evidence into a national injury prevention strategy. J Public Health.

[17] Rowan's Law (Concussion Safety) (2018). Bill 193.

[18] Government of Canada (2017). Department of Justice. Cannabis legalization and regulation.

[19] Baker EL, Potter MA, Jones DL, Mercer SL, Cioffi JP, Green LW (2005). The public health infrastructure and our nation's health. Annu Rev Public Health.

[20] Gebbie KM, Turnock BJ (2006). The public health workforce, 2006: new challenges. Health Aff (Millwood).

[21] Jacob RR, Baker EA, Allen P, Dodson EA, Duggan K, Fields R (2014). Training needs and supports for evidence-based decision making among the public health workforce in the United States. BMC Health Serv Res.

[22] Baillie E, Bjarnholt C, Gruber M, Hughes R (2009). A capacity-building conceptual framework for public health nutrition practice. Public Health Nutr.

[23] Stark CM, Graham-Kiefer ML, Devine CM, Dollahite JS, Olson CM (2011). Online course increases nutrition professionals' knowledge, skills, and self-efficacy in using an ecological approach to prevent childhood obesity. J Nutr Educ Behav.

[24] Khenti A, Freel S, Trainor R, Mohamoud S, Diaz P, Suh E (2016). Developing a holistic policy and intervention framework for global mental health. Health Policy Plan.

[25] Olley M (2007). Implementing a continuing education strategy to advance practice and practitioner development within an infection control service. Br J Infect Control.

[26] Lysyk B (2017). Auditor general report annual report.

[27] Lord S, Tator CH, Wells S (2010). Examining Ontario deaths due to all-terrain vehicles, and targets for prevention. Can J Neurol Sci.

[28] Panel on Prevention of Falls in Older Persons (2011). Summary of the updated American Geriatrics Society/British geriatrics society clinical practice guideline for prevention of falls in older persons. J Am Geriatr Soc.

[29] Canadian Council of Motor Transportation Administrators (2016). Canada's Road Safety Strategy 2025. Towards Zero: The Safest Roads in the World.

[30] DeCorby-Watson K, Mensah G, Bergeron K, Abdi S, Rempel B, Manson H (2018). Effectiveness of capacity building interventions relevant to public health practice: a systematic review. BMC Public Health.

[31] DeGroff A, Schooley M, Chapel T, Poister TH (2010). Challenges and strategies in applying performance measurement to federal public health programs. Eval Program Plann.

[32] Public Health Agency of Canada (2016). Canadian Best Practices Portal. The Organising Framework of the Public Health Approach.

[33] Mitchell RJ, Ryder T (2020). Rethinking the public health model for injury prevention. Inj Prev.

[34] Hanson DW, Finch CF, Allegrante JP, Sleet D (2012). Closing the gap between injury prevention research and community safety promotion practice: revisiting the public health model. Public Health Rep.

