# Supplementary material for: How can we support best practice? A situational assessment of injury prevention practice in public health
Source: BMC Public Health. 2020 Apr 3;20:431. doi: 10.1186/s12889-020-08514-x (PMC7119282; doi:10.1186/s12889-020-08514-x)
Supplement: Supplementary file 1 — Additional file 1: Table S1. Key informant and focus group interview guides. [file 12889_2020_8514_MOESM1_ESM.docx]

**Supplementary Table 1.** Key informant and focus group interview guides

| **Key Informant Questions** |
| --- |
| 1. How would you describe the current state of injury prevention practice in Ontario? |
| 2. What opportunities and challenges exist in addressing the public health burden of injuries in Ontario? |
| 3. What would you say are the greatest needs for successful injury prevention practice? |
| 4. How do you think that Public Health Ontario can support public health practice in injury?  *Probes*: Data, evidence synthesis, training, research, collaboration  a) What injury prevention topic support is needed (e.g., off-road vehicle safety or sub topic)  b) What kind of support is needed? (e.g., format – training, workshop). |
| 5. Who else do you think I should speak to? |
| **Focus Group Questions (Front Line Staff)** |
| 1. Can you describe the context of injury prevention programming work related to addressing injury issues in your health unit?  *Probes*: How the health unit develops and delivers programming  a) Who is involved? How are decisions made?  b) How is this process similar and/or different to other program areas? |
| 2. What supports would assist the planning and implementation of your programming?  *Probes*: How would these supports be best delivered or disseminated? |
| 3. What resources do you have to do this work?  *Probes*: Funding, tools, partnerships, the ‘right’ expertise and/or knowledge |
| 4. What are the top three injury issues for your health unit? (both current and emerging) |
| 5. What are the top challenges you face when working on these issues?  *Probes*: In the context of scientific and technical guidance  a) Tell me more about how more/better [item] would lead to improvements in your programming?  b) What difference do you think it would make to your programming?  c) Is there an example where this kind of more/better [item] led to program improvement?  d) How do you see collaborating with Public Health Ontario on some of these challenges? |
