# Supplementary material for: How can we support best practice? A situational assessment of injury prevention practice in public health
Source: BMC Public Health. 2020 Apr 3;20:431. doi: 10.1186/s12889-020-08514-x (PMC7119282; doi:10.1186/s12889-020-08514-x)
Supplement: Supplementary file 2 — Additional file 2: Table 2. Consolidated criteria for reporting qualitative studies (COREQ) checklist1. [file 12889_2020_8514_MOESM2_ESM.docx]

| **Supplementary Table 2**. Consolidated criteria for reporting qualitative studies (COREQ) checklist^1^ | | |
| --- | --- | --- |
| **No. Item** | **Guide Questions/Description** | **Reported on Page #** |
| **Domain 1: Research Team and Reﬂexivity** | | |
| *Personal Characteristics* | | |
| 1. Interviewer/facilitator | Which author/s conducted the interview or focus group? | Page 4 |
| 2. Credentials | What were the researcher’s credentials? e.g. PhD, MD | Page 1 |
| 3. Occupation | What was their occupation at the time of the study? | Page 1 |
| 4. Gender | Was the researcher male or female? | Page 1 |
| 5. Experience and training | What experience or training did the researcher have? | Page 1, 4 |
| *Relationship with Participants* | | |
| 6. Relationship established | Was a relationship established prior to study commencement? | Page 4 |
| 7. Participant knowledge of the interviewer | What did the participants know about the researcher? e.g. personal goals, reasons for doing the research | Page 5 |
| 8. Interviewer characteristics | What characteristics were reported about the interviewer/facilitator? e.g. bias, assumptions, reasons and interests in the research topic | Page 5, 12 |
| **Domain 2: Study Design** | | |
| *Theoretical Framework* | | |
| 9. Methodological orientation  and theory | What methodological orientation was stated to underpin the study? e.g. grounded theory, discourse analysis, ethnography, phenomenology, content analysis | Page 4 |
| *Participant Selection* | | |
| 10. Sampling | How were participants selected? e.g. purposive, convenience, consecutive, snowball | Page 4 |
| 11. Method of approach | How were participants approached? e.g. face-to-face, telephone, mail, email | Page 4 |
| 12. Sample size | How many participants were in the study? | Page 6 |
| 13. Non-participation | How many people refused to participate or dropped out? Reasons? | Page 6 |
| *Setting* | | |
| 14. Setting of data collection | Where was the data collected? e.g. home, clinic, workplace | Page 4 |
| 15. Presence of non-participants | Was anyone else present besides the participants and researchers? | Page 5 |
| 16. Description of sample | What are the important characteristics of the sample? e.g. demographic data, date | Page 4 |
| *Data Collection* | | |
| 17. Interview guide | Were questions, prompts, guides provided by the authors? Was it pilot tested? | Supplementary table 1 |
| 18. Repeat interviews | Were repeat interviews carried out? If yes, how many? | Page 4-5 |
| 19. Audio/visual recording | Did the research use audio or visual recording to collect the data? | Page 4-5 |
| 20. Field notes | Were field notes made during and/or after the interview or focus group | Page 4-5 |
| 21. Duration | What was the duration of the interviews or focus group? | Page 4 |
| 22. Data saturation | Was data saturation discussed? | Page 5 |
| 23. Transcripts returned | Were transcripts returned to participants for comment and/or correction? | Page 6 |
| **Domain 3: Analysis and Findings** | | |
| *Data Analysis* | | |
| 24. Number of data coders | How many data coders coded the data? | Page 5 |
| 25. Description of the coding tree | Did authors provide a description of the coding tree? | Page 5 |
| 26. Derivation of themes | Were themes identified in advance or derived from the data? | Page 5 |
| 27. Software | What software, if applicable, was used to manage the data? | N/A |
| 28. Participant checking | Did participants provide feedback on the findings? | Page 6 |
| *Reporting* | | |
| 29. Quotations presented | Were participant quotations presented to illustrate the themes/findings? Was each quotation identified? e.g. participant number | N/A |
| 30. Data and findings consistent | Was there consistency between the data presented and the findings? | Page 10-11 |
| 31. Clarity of major themes | Were major themes clearly presented in the findings? | Page 7-8 |
| 32. Clarity of minor themes | Is there a description of diverse cases or discussion of minor themes? | Table 2, Page 7-8 |
| ^1.^ Tong A, Sainsbury P, Craig J. Consolidated criteria for reporting qualitative research (COREQ): a 32-item checklist for interviews and focus groups. *Int J Qual Health Care* 2007; 19(6):349 – 357 | | |
